# Mifepristone inhibits ovarian cancer metastasis by intervening in SDF-1/CXCR4 chemokine axis

**DOI:** 10.18632/oncotarget.19289

**Published:** 2017-07-17

**Authors:** Ning Zheng, Jiahang Chen, Weiqun Liu, Jian Liu, Tao Li, Hongning Chen, Jichuang Wang, Lee Jia

**Affiliations:** ^1^ Cancer Metastasis Alert and Prevention Center, and Biopharmaceutical Photocatalysis, State Key Laboratory of Photocatalysis on Energy and Environment, Fuzhou University, Fuzhou 350002, China; ^2^ Fujian Provincial Key Laboratory of Cancer Metastasis Chemoprevention and Chemotherapy, Fuzhou University, Fuzhou 350002, China; ^3^ Fujian Key Laboratory for Translational Research in Cancer and Neurodegenerative Diseases, Institute for Translational Medicine, Fujian Medical University, Fuzhou 350108, China

**Keywords:** mifepristone (RU486), the SDF-1/CXCR4 chemokine axis, ovarian cancer, cancer metastasis, actin polymerization

## Abstract

SDF-1/CXCR4 signaling axis determines the proliferative potential and site-specific cancer metastasis. Recent studies suggest involvement of the axis and steroidal hormone in ovarian cancer metastasis. Here we hypothesize that mifepristone (RU486), a well-known progesterone-based abortifacient, might interfere this axis and inhibit ovarian cancer metastasis. Mifepristone at concentrations < IC50 inhibited expression of CXCR4 on cell surface of ovarian cancer SKOV-3 and IGROV-1, and reduced expression of the intracellular CXCR4 protein and its related mRNA activated by SDF-1. SDF-1 significantly stimulated proliferation of SKOV-3 and IGROV-1 cells with concomitant increases in intracellular phosphorylation of Akt and ERK. SDF-1 activated cell chemotatic migration and actin polymerization, and up-regulated expression of MMP-2, MMP-9, COX-2, VEGF without influencing the adhesion molecules ICAM-1 and integrins β1, α1, α3, α5, and α6. The above-mentioned effects of SDF-1 could be antagonized by mifepristone concentration-dependently, and CXCR4 antagonist AMD3100. Mifepristone suppressed the SDF-1-induced migration, invasion and adhesion of the cancer cells to extracellular matrixes. Three-day pretreatment of nude mice with mifepristone (5 and 20 mg/kg/day) followed by a single intraperitoneal IGROV-1 inoculation, along with repeated SDF-1 and mifepristone administrations in turn every other day for 36 days significantly reduced ascitic fluid, metastatic foci, tumor weight and immunoreactivity of CXCR4 in comparison with the SDF-1-treated control. Our results suggest that mifepristone inhibit SDF-1/CXCR4 signaling axis, may have preventive and therapeutic effects on ovarian cancer metastasis.

## INTRODUCTION

With an overall survival rate of less than 30%, ovarian cancer remains a poorly addressed health problem relative to other generally diagnosed gynecologic malignancies. The vast majorities of patients with ovarian cancer, particularly epithelial ovarian cancer, are diagnosed at the advanced stages (stages III and IV) with disseminated intraperitoneal carcinomatosis, and ultimately succumb to metastatic disease [1].

Chemokines or chemotactic cytokines with a molecular mass around 8-12 kDa belong to a superfamily that direct cell trafficking of a wide variety of cell types. Chemokines binding to corresponding specific chemokine receptors induce leukocyte infiltration, regulate immune functions, conduct the homing of carcinomas cells to specific metastatic sites, and mediate angiogenesis at the tumor microenvironment [2]. Dysregulated expression of the chemokine ligand SDF-1 (stromal derived factor-1, or CXCL12), or its cognate receptor CXCR4 (C-X-C chemokine receptor type 4), is associated with higher grades and poorer prognosis in various human cancers, including ovarian carcinomas [3]. Targeted metastasis of cancer cells with elevated expression of CXCR4 to specific sites guided by the high level of SDF-1 suggests a new therapeutic strategy to interrupt the SDF-1/CXCR4 axis and inhibit cancer metastasis [4].

In addition, many evidences show that SDF-1/CXCR4 signaling pathway plays a crucial role during embryogenesis [5–7]. Steroid hormones are capable of increasing the CXCR4 expression in mRNA levels and protein levels in human endometrial stromal cells for implantation and pregnancy [8]. The long-term administration of oral contraceptives could improve the survival of cancer patients [9], due to the biological similarities between tumor metastases and embryonic implantation [10–13]. As a synthetic steroid compound, mifepristone (RU486) is initially used to terminate pregnancy in the first month in clinic [14]. Recently, a mass of preclinical and clinical studies confirm that mifepristone has prominent anti-tumor effects on a wide range of tumor types, such as gastric, breast, prostate, endometrial and ovarian adenocarcinoma [15, 16]. However, the anti-metastatic activity of mifepristone remains poorly understood, and the precise molecular mechanisms are still emerging.

Therefore, we hypothesized that mifepristone may suppress the expression and function of SDF-1/CXCR4 signaling axis, and further interfere this chemokine axis with concomitant inhibition of cancer metastasis. To test the hypothesis, the effects of mifepristone on CXCR4 expression, SDF-1/CXCR4 interaction and downstream signaling events were evaluated. Notably, we explored the underlying mechanism by which mifepristone inhibited ovarian cancer metastasis.

## RESULTS

### Mifepristone decreased CXCR4 expression in ovarian cancer cells

**Figure 1:**
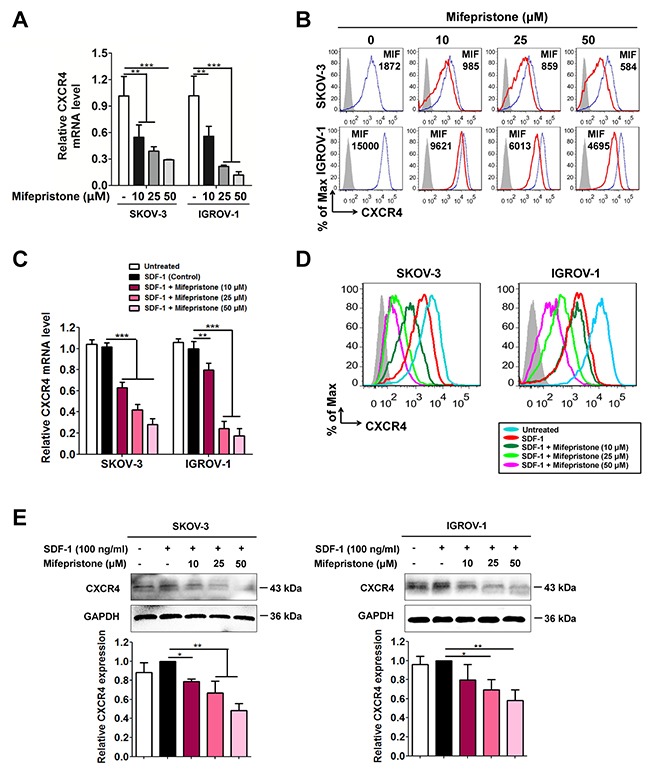
Mifepristone decreases the expression levels of CXCR4 in a concentration-dependent manner in SKOV-3 and IGROV-1 cells Mifepristone at concentrations < IC50 down-regulated the CXCR4 expression at mRNA levels **(A)**, and on cell surface of ovarian cancer SKOV-3 and IGROV-1 which was measured by mean fluorescence intensity (MFI) **(B)**. **(C)** SDF-1 treatment was unable to change CXCR4 mRNA levels, but mifepristone suppressed CXCR4 mRNA levels concentration-dependently. **(D)** The functional CXCR4 expression on the cell surface was suppressed by SDF-1 treatment, and further down-regulated by mifepristone in the presence of SDF-1. (E) the total protein level of CXCR4 was not altered by SDF-1 stimulation, but significantly reduced by mifepristone treatment in the presence of SDF-1.*, *P* < 0.05; **, *P* < 0.01; ***,*P* < 0.001. Values = Mean ± SD. *n* = 3.

Using the RT-PCR analysis, we first showed that mifepristone triggered a concentration-dependent decrease in CXCR4 expression at the levels of mRNA in SKOV-3 and IGROV-1 cells (Figure 1A). Likewise, a significant decrease in the functional CXCR4 expression on the cell surface was observed in the cells treated with mifepristone when compared with the untreated cells (Figure 1B).

As CXCR4 was activated by SDF-1, we found no change in CXCR4 expression at the levels of mRNA and total protein (Figure 1C and 1E), but SDF-1 itself was able to produce a significant decline of CXCR4 expression on the cell surface (Figure 1D). The CXCR4 expression at the levels of mRNA, cell surface proteins and total proteins was all down-regulated by mifepristone in the presence of SDF-1 (Figure 1C-1E).

### Mifepristone inhibited the SDF-1/CXCR4-mediated downstream cell signaling and cell proliferation of ovarian cancer

**Figure 2:**
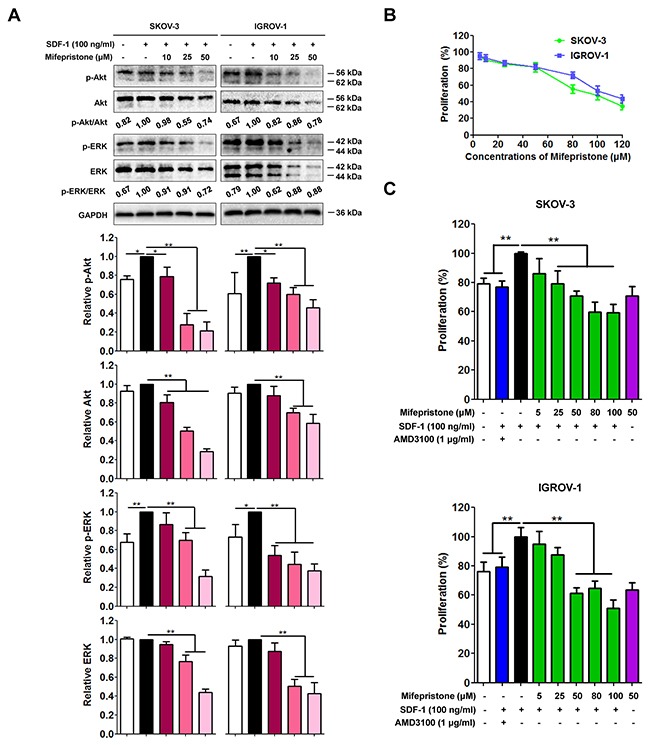
Mifepristone inhibits the SDF-1/CXCR4-mediated downstream cell signaling and cell proliferation in SKOV-3 and IGROV-1 cells **(A)** SDF-1 was incapable of altering expression of Akt and ERK, but promoted Akt and ERK phosphorylation. Mifepristone suppressed the expression of Akt, ERK, p-Akt and p-ERK in a concentration-dependent manner, resulting in the decline of the p-Akt/Akt and p-ERK/ERK ratios. The immunoblot images and the related quantitative analysis were showed on the upper panel and lower panel, respectively. **(B)** Mifepristone alone produced the significant inhibition of the growth of SKOV-3 and IGROV-1 cells after 24-hour treatment. **(C)** Mifepristone inhibited SDF-1-enhanced proliferation of the cancer cells. However, there was no significant difference in inhibition of ovarian cancer growth between mifepristone alone (50 μM) and mifepristone in the presence of SDF-1. The SDF-1 effect was antagonized by AMD3100. *, *P* < 0.05; **, *P* < 0.01. Values = Mean ± SD. *n* = 3.

We next predicted that mifepristone may decrease activity of Akt and ERK. Immunoblot analysis showed that SDF-1 didn't change the Akt and ERK protein levels, but significantly increased the expression of Akt and ERK phosphorylation (Figure 2A). The expression of total Akt, p-Akt, total ERK and p-ERK were all significantly decreased by mifepristone concentration-dependently, resulting in the decline of the p-Akt/Akt and p-ERK/ERK ratios in mifepristone-treated cells (Figure 2A).

We also performed the functional studies to investigate whether mifepristone inhibited SDF-1/CXCR4-mediated cellular functions. Mifepristone alone produced the significant inhibition of the growth of SKOV-3 and IGROV-1 cells after 24-hour treatment (Figure 2B). The IC50 values of mifepristone were 91.93 ± 10.21 μM for SKOV-3 cells and 113.80 ± 17.19 μM for IGROV-1 cells (Figure 2B). SDF-1 may facilitate tumor growth in an autocrine or paracrine fashion by directly stimulating cells survival and proliferation via CXCR4 receptor [17]. Using the MTT assay, we tested a mechanistic link between mifeprsitone and suppression of cancer cells proliferation through the SDF-1/CXCR4 axis. There was no significant difference in inhibition of ovarian cancer growth between mifepristone alone (50 μM) and mifepristone in the presence of SDF-1 (Figure 2C). AMD3100 (1 μg/ml), a well-documented CXCR4 antagonist, inhibited cell proliferation in the presence of SDF-1, and verified the function of the SDF-1/CXCR4 axis in the tested ovarian cancer cells. These results suggest that mifepristone block SDF-1/CXCR4-mediated cell proliferation and its related cell signaling in ovarian cancer cells.

### Mifepristone inhibited SDF-1-stimulated cell migration

**Figure 3:**
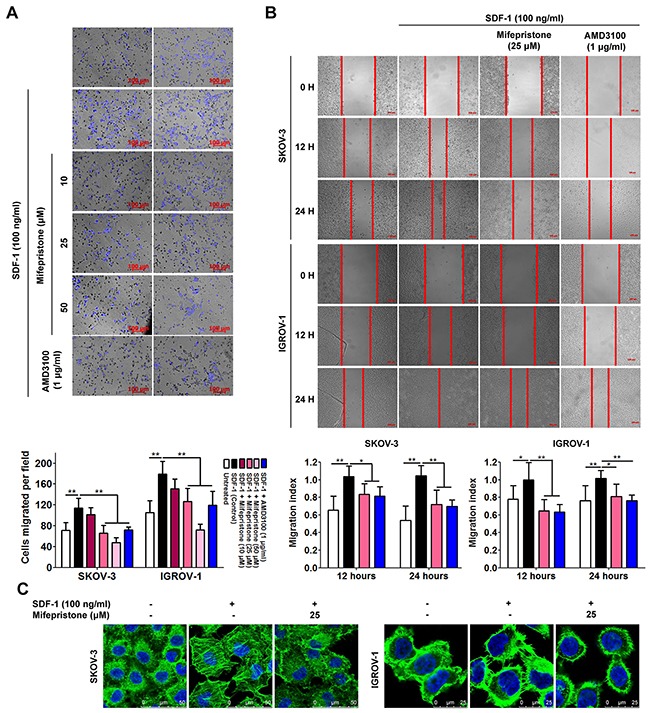
Mifepristone attenuates SDF-1-facilitated cell migration and actin polymerization in SKOV-3 and IGROV-1 cells Mifepristone inhibited SDF-1-stimulated chemotatic migration **(A)** and cell mobility **(B)** in both SKOV3 and IGROV1 cells. The microscopic images and the related quantitative analysis were showed on the upper panel and lower panel, respectively. **(C)** More stressed actin fibers and filopodia was produced in the SDF-1-stimulated cells, whereas, the mifepristone-treated cells were rounded primarily in cortical bundles and exhibited less stressed actin fibers. The SDF-1 effect was antagonized by AMD3100. *, *P* < 0.05; **, *P* < 0.01. Values = Mean ± SD. *n* = 3.

SDF-1 alone significantly accelerated chemotatic migration (Figure 3A). However, pretreatment with mifepristone reversed the SDF-1-induced migration. The upper panel of Figure 3A shows the microscopic images of cell migration in the presence and absence of SDF-1, mifepristone and AMD3100. The lower panel shows the quantitative analysis of these data. Next, we verified this result using the wound-healing assay. The wound healing of SKOV-3 and IGROV-1cells was significantly increased by SDF-1 stimulation (Figure 3B). Whereas, mifpristone elicited the significant inhibition on the SDF-1-stimulated wound healing (Figure 3B). AMD3100 could antagonize the above-mentioned effect of SDF-1 (Figure 3A and 3B), suggesting the involvement of the SDF-1/CXCR4 axis in the cell movement and the effect of mifepristone on the movement.

Last, we questioned whether the assembly of cell migration machinery was influenced by mifepristone. Immunofluorescence microscopy revealed that SKOV-3 cells were rounded with few cytoplasmic projections, and showed a typical epithelial-like morphology, which was consistent with the non-migration phenotype in the absence of any stimulation (Figure 3C). By comparison, SKOV-3 cells stimulated with 100 ng/ml of SDF-1 showed a higher percentage of actin stress fibers with more spreading (Figure 3C). Particularly, mifepristone decreased formation of actin stress fibers and cell spreading (Figure 3C). Without any stimulation, IGROV-1 cells posed an epithelial-like morphology with disorganized cytoskeleton (Figure 3C). F-actin in the SDF-1-treated IGROV-1 cells was accumulated and reorganized with abundant filopodia and lamellopodia (Figure 3C). Treatment with mifepristone reversed SDF-1-stimulated actin polymerization with organized primarily in cortical bundles (Figure 3C).

Taken together, these data suggest that mifepristone could inhibit chemotatic migration in a concentration-dependent manner (Figure 3A and 3B). The prevention of F-actin polymerization by mifepristone led to the loss of actin stress fibers and filopodia, and the accumulation of cortical actin bundles (Figure 3C).

### Mifepristone inhibited SDF-1-stimulated cell invasion and MMP-2, MMP-9, COX-2 and VEGF expression

**Figure 4:**
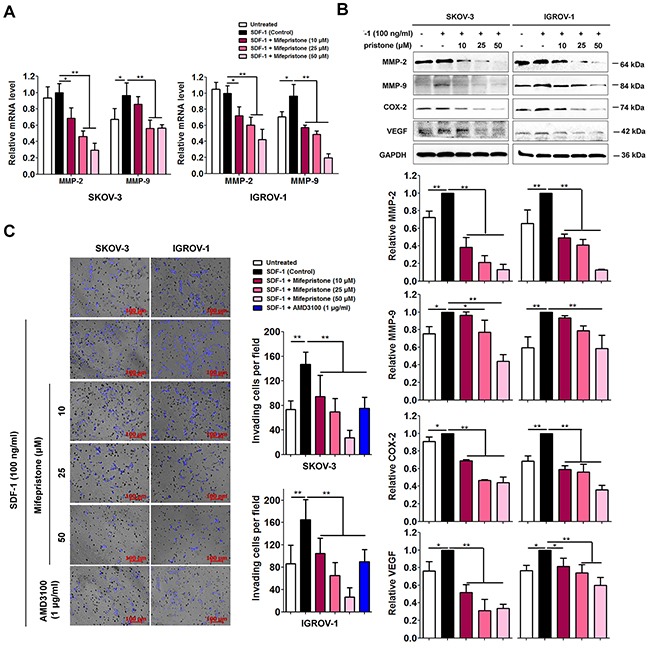
Mifepristone reduces expression of MMP-2, MMP-9, COX-2 and VEGF, and the related cell invasion in SKOV-3 and IGROV-1 cells **(A)** Mifepristone suppressed SDF-1-increased MMP-2 and MMP-9 mRNA levels. **(B)** Mifepristone down-regulated SDF-1-elevated protein levels of MMP-2, MMP-9, COX-2 and VEGF in a concentration-dependent manner. The immunoblot images and the related quantitative analysis were showed on the upper panel and lower panel, respectively. **(C)** Mifepristone inhibited SDF-1-promoted cell invasion. The SDF-1 effect was antagonized by AMD3100. *, *P* < 0.05; **, *P* < 0.01. Values = Mean ± SD. *n* = 3.

The activation and overexpression of MMP-2 and MMP-9 correlate with the SDF-1/CXCR4-stimulation [18]. We assessed the expression levels of MMP-2 and MMP-9 in mifepristone-treated ovarian cancer cells. By the RT-PCR and immunoblot analyses, we found mifepristone elicited a significant inhibition in SDF-1-increased MMP-2 and MMP-9 expression at both the mRNA and protein levels in the two cell lines (Figure 4A and 4B).

MMPs, COX-2 and VEGF belong to key angiogenesis factors to accelerate pathological angiogenesis associated with tumor and the subsequent cancer metastasis [19]. SDF-1 elevated COX-2 and VEGF protein levels, but this elevation was inhibited in a concentration-dependent manner after mifepristone treatment evidenced by the immunoblot analysis (Figure 4B).

As previous studies demonstrated that SDF-1-induced invasiveness is depended on CXCR4 binding [18], we asked whether mifepristone was capable of intervening in cell invasion through CXCR4 down-regulation. The transwell invasion assay showed that the cells in the absence of mifepristone pre-incubation penetrated Matrigel and migrated toward SDF-1, whereas the cells pre-treated with mifepristone showed slow migration in response to chemokine stimulation (Figure 4C). AMD3100 could antagonize the above-mentioned effect of SDF-1.

### Mifepristone diminished SDF-1-induced adhesion to fibronectin and matrigel

**Figure 5:**
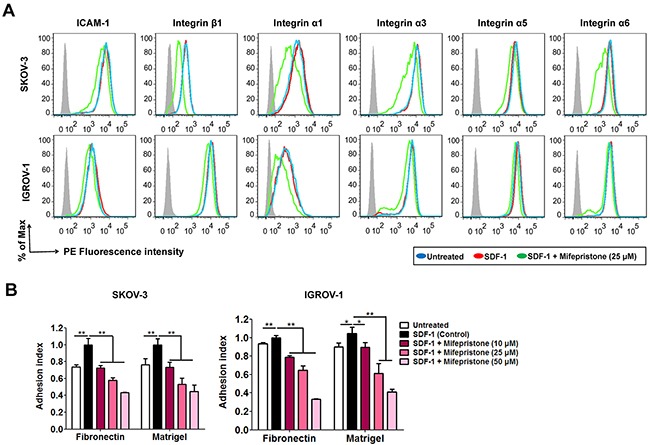
Mifepristone inhibits the surface expression of adhesion molecules, and SDF-1-induced cell adhesion in SKOV-3 and IGROV-1 cells **(A)** Mifepristone down-regulated the surface expression of ICAM-1 and integrins β1, α1, α3, α5 and α6, whereas SDF-1 had no effect on them. **(B)** Mifepristone inhibited SDF-1-enhanced adhesion of SKOV-3 and IGROV-1 cells to fibronectin and Matrigel. *, *P* < 0.05; **, *P* < 0.01. Values = Mean ± SD. *n* = 3.

SDF-1 increases adhesion of tumor cells to the extracellular matrices or endothelium cells by activating or modulating the function of several cell surface integrins [20]. By using the flow cytometry analysis, we did not observe any alter in the expression levels of ICAM-1, integrin β1, integrin α1, integrin α3, integrin α5 and integrin α6 on the cell surface of both SKOV-3 and IGROV-1 cells after stimulation with SDF-1 for 24 hours (Figure 5A). However, mifepristone decreased the expression of these adhesion molecules on the cell surface, except for integrin α5 and integrin α6 in IGROV-1 cells (Figure 5A). In addition, mifepristone attenuated SDF-1-enhanced adhesion of ovarian cancer cells to fibronectin and Matrigel (Figure 5B).

### Mifepristone blocked SDF-1-facilitated ovarian cancer peritoneal metastasis

**Figure 6:**
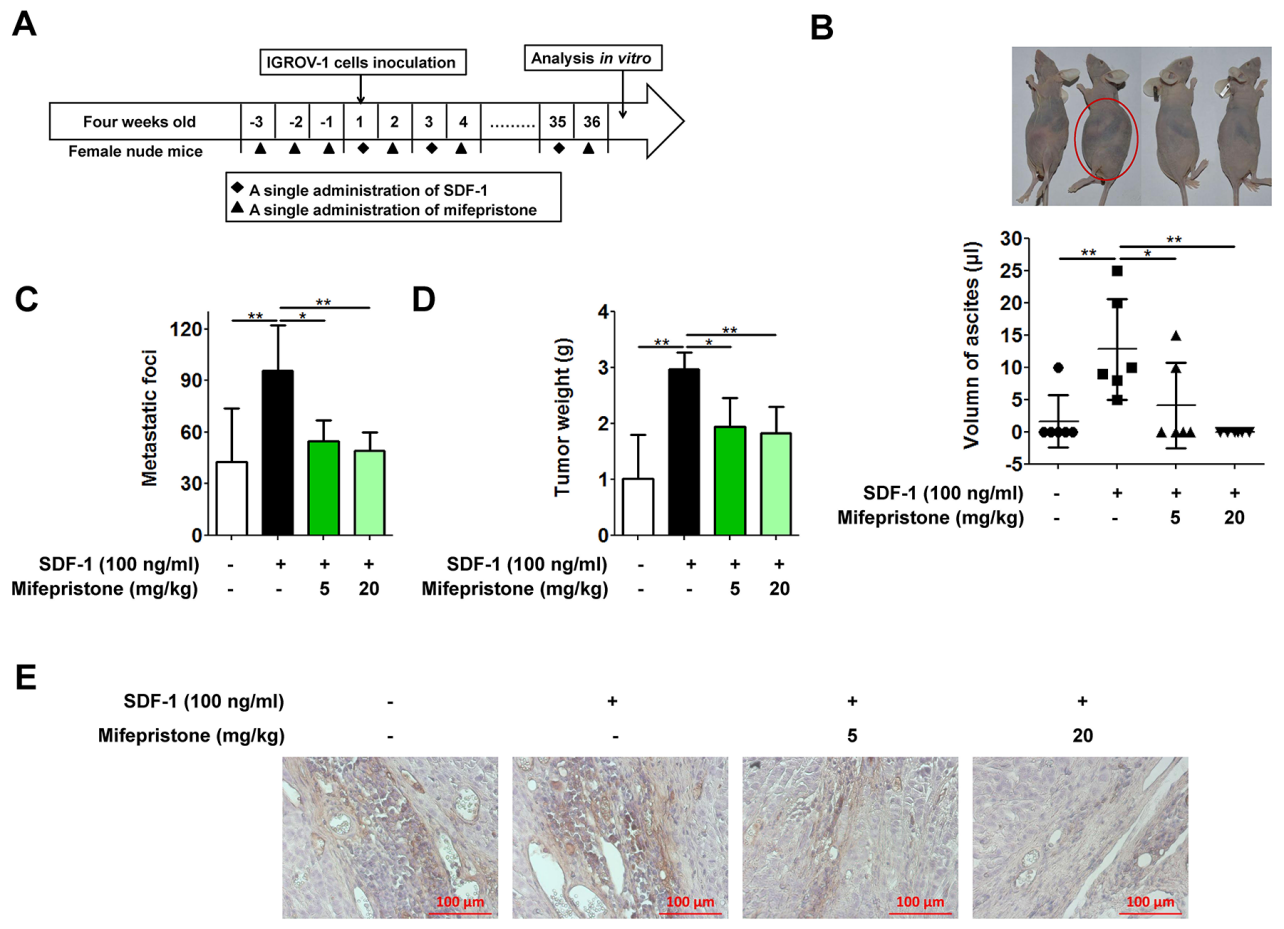
Mifepristone suppresses peritoneal metastasis of human ovarian cancer cells in the nude mice **(A)** Three-day pretreatment of nude mice with mifepristone (5 or 20 mg/kg) followed by IGROV-1 inoculation, along with repeated mifepristone and SDF-1 administrations in turn every other day for 36 days significantly decreased malignant ascites **(B)**, metastatic lesions **(C)**, tumor weight **(D)** and CXCR4 expression **(E)** when compared with the SDF-1-treated nude mice. We separated and combined the visible tumor nodules in each nude mouse to represent the tumor weight. *, *P* < 0.05; **, *P* < 0.01. Values = Mean ± SD. *n* = 6.

The effect of mifepristone on ovarian cancer peritoneal metastasis was performed in the nude mice as described in Figure 6A, and the Materials and Methods. The untreated and mifepristone-treated mice both showed less malignant ascites when compared with the mice treated with SDF-1 alone (Figure 6B). In addition, the mifepristone-treated mice showed fewer and smaller metastatic nodules than the mice with SDF-1 treatment (Figure 6C), evidenced by the lighter tumor weight (Figure 6D). Consistent with the *in vitro* assay, mifepristone was able to down-regulate the CXCR4 expression of tumor tissue by immunohistochemical analysis, when compared with the untreated and SDF-1-treated mice (Figure 6E). The above results reveal the threrapeutic effect of mifepristone on ovarian cancer peritoneal metastasis.

## DISCUSSION

Overexpression of CXCR4 has become an independent prognostic factor of poor survival in human epithelial ovarian cancer [21]. The safety and efficacy of intervention into the SDF-1/CXCR4 signaling axis is required for ovarian carcinogenesis metastatic prevention and therapy. At present, the pharmacological intervention was primarily small molecular antagonists of CXCR4; for instance, AMD3100 and Plerixafor. However, a mass of clinical data suggest these conventional CXCR4 antagonists fail to provide therapeutic benefits for patients with tumors or HIV. The failure was attributed to their chronic toxicity, short *in vivo* half-lives, poor oral bioavailability and so on [22]. Mifepristone (RU486) has been used commonly as a contraceptive by millions of women worldwide. Recently, mifepristone was also used for cancer clinical trials by both genders [23]. The present study was the first to observe the changes of SDF-1/CXCR4 axis in cancer cells with mifepristone treatment. While other studies focused only on the role of chemokines or mifepristone in tumor development, we revealed the function of mifepristone on tumor progression and metastasis via interfering with the SDF-1/CXCR4 axis.

Using the RT-PCR, immunoblot and flow cytometric analyses, we revealed that mifepristone elicited a concentration-dependent decrease in CXCR4 expression at the levels of mRNA, cell surface proteins and total proteins at the presence and absence of SDF-1 (Figure 1). As downstream signalings of the SDF-1/CXCR4 chemokine axis, Akt and ERK were CXCR4-dependent cell survival factors [24]. Through suppression of CXCR4 expression, mifepristone down-regulated the intracellular expression of Akt, ERK, p-Akt and p-ERK in ovarian cancer cell lines (Figure 2A). The overall reduction in the ratios of p-Akt/Akt and p-ERK/ERK resulted in the inhibition by mifepristone of cell viability in the presence and absence of SDF-1 (Figure 2).

Actin polymerization to form stress fibers or pseudopodia meets the requirement for cancer cell spreading and invasiveness [25]. SDF-1 facilitated reorganization of F-actin to produce abundant stress fibers, filopodia and lamellopodia, and the resultant enhancement of cell movement (Figure 3). In comparison, mifepristone successfully suppressed cell mobility by cytoskeletal dysregulation (Figure 3). Cancer cells secrete MMPs (eg, MMP-2 and MMP-9) to degrade the extracellular matrices [26]. COX-2 promotes the secretion and release of MMP-2 and MMP-9 to involve in tumor progression and metastasis [27]. VEGF, as a well-known angiogenic factor, facilitates angiogenesis in tumor [28]. In addition, MMPs, COX-2 and VEGF are important members of the downstream signaling pathways of the SDF-1/CXCR4 axis. SDF-1 stimulates the invasion of ovarian cancer cells into the peritoneal cavity in the CXCR4-dependent manner [29]. The data showed that SDF-1 treatment elevated the protein levels of MMP-2, MMP-9, COX-2 and VEGF with concomitant more invasive capability (Figure 4). In contrast, mifepristone significantly reduced the SDF-1-enhanced MMP-2, MMP-9, COX-2 and VEGF expression and successfully inhibited cell invasion (Figure 4). The reduction by mifepristone of adhesive capacity of ovarian cancer cells to fibronectin and Matrigel was attributed to the suppression of the activation by SDF-1 of adhesion molecules and the decline in the cell surface expression of functional adhesion molecules (Figure 5).

In the xenograft tumor model, SDF-1/CXCR4 axis not only promoted homing and colonization of cancer cells to a distant tissue, but also enhanced the growth of secondary tumor. Mifepristone administration intraperitoneally remarkably inhibited cancer cells spreading and proliferation in the new metastatic organs that were reflected in the volume of ascites, metastatic foci and tumor weight (Figure 6B-6D). In addition, mifepristone effectively reduced the CXCR4 expression, evidenced by lower CXCR4 immunoreactivity in comparison with the mice of the untreated and SDF-1-treated alone (Figure 6E).

**Figure 7:**
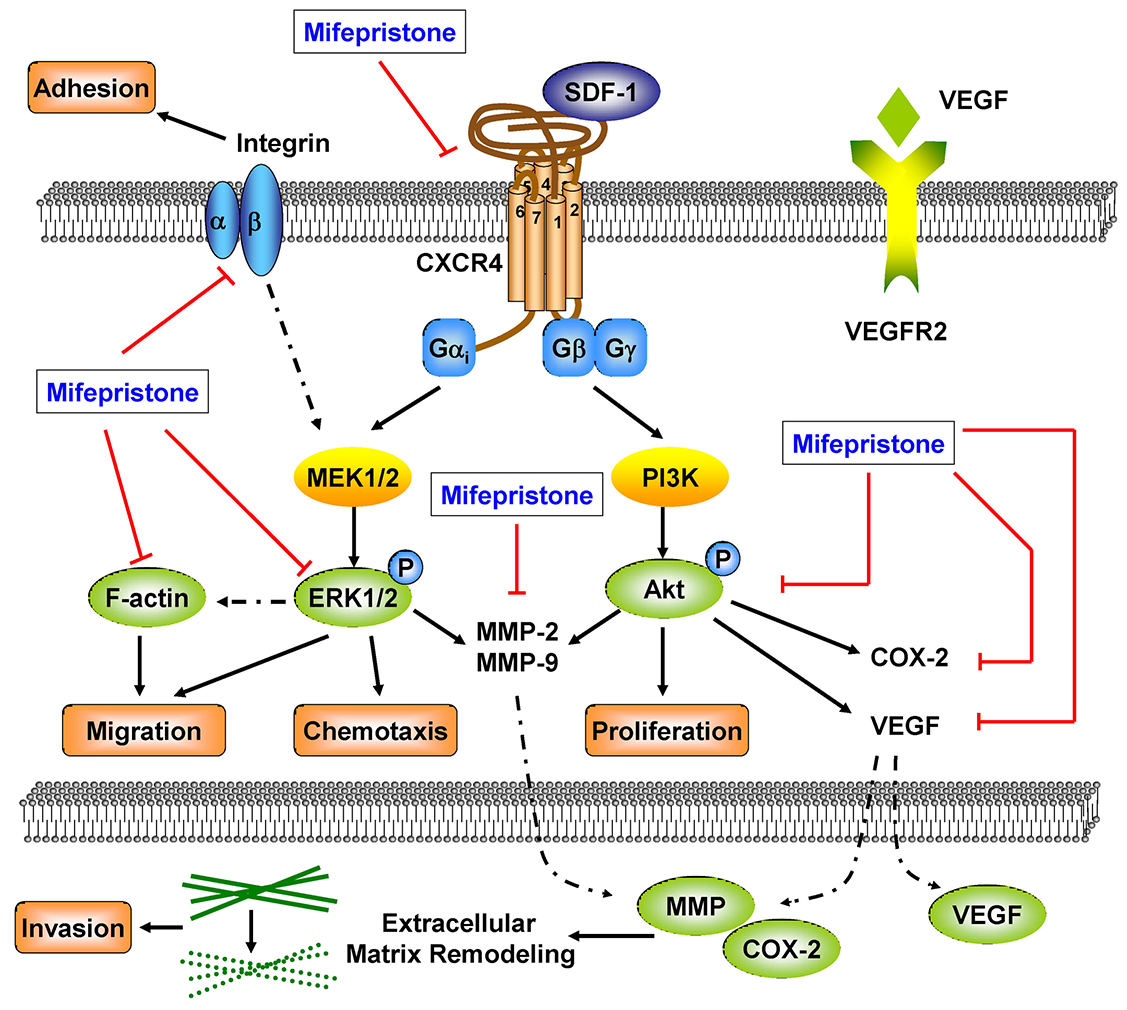
The underlying mechanisms involved in the suppression of caner metastasis by mifepristone

In the present study, the abortifacient mifepristone was demonstrated to be a novel chemokine receptor CXCR4 blocking agent. Mifeprisotne could down-regulate cellular CXCR4 expression, interrupt the SDF-1/CXCR4 interaction and block the related downstream signaling pathways. The data suggested mifepristone treatment success in inhibiting cancer cell proliferation, adhesion, mobility and invasiveness, resulting in the inhibition of cancer metastasis (Figure 7). The preventive and therapeutic effects of mifepristone on cancer metastasis deserve further exploration and clinical trials.

## MATERIALS AND METHODS

### Reagents

Mifepristone was provided by Shanghai New Hualian Pharmaceutical Co. with purity > 98%. Recombinant human CXCL12 was from Sino Biological Inc. (Beijing, China). AMD3100 was obtained from Sigma (St. Louis, MO, USA). Alexa Fluor^®^ 488 phalloidin (#A12379) was purchased from Invitrogen (Carsbad, CA, USA). The following antibodies were used: anti-CXCR4 (sc-9046), anti-Akt 1/2/3 (sc-8312), anti-p-Akt 1/2/3 (Ser 473, sc-33437), anti-ERK 1/2 (sc-135900), anti-p-ERK 1/2 (Thr 202/Tyr 204, sc-16982), anti-VEGF (sc-152), and secondary horseradish peroxidase-conjugated anti-rabbit (sc-2054) and anti-mouse (sc-2031) from Santa Cruz Biotechnology, Inc. (Santa Cruz, CA, USA); anti-MMP-2 (#13132), anti-MMP-9 (#13667) and anti-COX-2 (#12282) from Cell Signaling Technologies (Beverly, MA, USA); anti-GAPDH (#AG019) from Beyotime Biotechnology (Haimen, China). The antibodies used in flow cytometric analysis were all from BD PharMingen (San Diego, CA, USA).

### Cell culture

The human epithelial ovarian cancer cell lines SKOV-3 and IGROV-1 were from the American Type Culture Collection (ATCC). Cell lines were routinely maintained in Dulbecco's modified Eagle's medium (DMEM) containing 10% (v/v) fetal bovine serum (FBS), 100 U/ml penicillin and 100 μg/ml streptomycin. The cells were incubated at 37°C in a humidified atmosphere containing 5% CO_2_.

### Cell proliferation assay

Cell proliferation was detected using MTT assay. Briefly, cells (6×10^3^ per well) were plated into a 96-well plate and cultured for 24 hours. After treatment with concentrations of mifepristone in the presence and absence of SDF-1 (100 ng/ml) or AMD3100 (1 μg/ml) for 24 hours, cells were incubated with 0.5 mg/ml MTT in the culture medium without phenol red for an additional 4 hours. The purple crystals were dissolved with 150 μl of DMSO. Finally, the 96-well plate was read on a microplate reader (Tecan, Hombrechtikon, Switzerland) at the test wavelength of 490 nm. The experiment was conducted in triplicate.

### Wound healing assay

Wound healing assay was performed to assess the effect of mifepristone on cell migration as we described previously [30]. Cells (2×10^4^ per well) were seeded onto a 24-well plate and cultured for 24 hours, the scratch was made across the cell monolayer with the tip. After washing 3 times with phosphate buffer solution (PBS), the serum-free medium containing 100 ng/ml of SDF-1 and mifepristone, or AMD3100 (1 μg/ml) was added to each well. Wound healing within the scrape line was recorded at 0, 12 and 24 hours after the scratching using a light microscope (Zeiss, Oberkochen, Germany). The migration index was used to evaluate the migration potential, which is the distance of migration in treated group compared with the distance of migration in control group.

### Transwell migration assay

Transwell migration assay was performed in the 6.5-mm-diameter chambers with 8-μm pore filters (Corning Costar, Cambridge, MA, USA) as we described previously [30]. After mifepristone or AMD3100 treatment for 24 hours, cells were suspended at 5×10^5^ cells/ml in the serum-free media, and the 0.2 ml cell suspension was added to the upper chamber. The 0.8 ml media containing 20% (v/v) FBS and 100 ng/ml of SDF-1 were placed to the lower chamber. Cells were allowed to migrate from the upper to lower chamber for 24 hours. The upper surface of the filters was scraped with cotton swabs to remove non-migrating cells. Then, the filters were fixed with methanol, and stained with DAPI (Roche, Basel, Switzerland). The number of migrating cells in six random high-power fields per filter was counted using a light microscope (Zeiss).

### Transwell invasion assay

The invasion assay was conducted as we described previously [31]. Except for the use of Matrigel, the transwell invasion assay shared a similar approach with the transwell migration assay. The non-invading cells were removed by scraping, and the invading cells that had permeated the Matrigel were fixed, stained and counted using a light microscope (Zeiss). Six representative fields of each insert were selected for examination.

### Cell adhesion assay

The assay was preformed as we described previously [32]. A 96-well plate was pre-coated with 30 μg/ml of fibronectin (BD Biosciences) or 10 μg/ml of Matrigel. Nonspecific binding sites were blocked with PBS containing 0.1% (w/v) bovine serum albumin (BSA) for 2 hours. After mifepristone treatment for 24 hours, cells (2.0×10^4^) resuspended in serum-free DMEM were allowed to adhere to the fibronectin- or Matrigel-coated wells for 1 hour in the presence and absence of 100 ng/ml of SDF-1. Unattached cells were removed, and the remaining adherent cells were evaluated using MTT assays as described previously. The adhesion index was used to evaluate the adhesion potential, which is the absorbance (490 nm) in the treated group compared with the absorbance in the control group.

### F-actin fluorescence microscopy

Cells were seeded on confocal dish and incubated in the serum-free medium containing SDF-1 (100 ng/ml) and mifepristone (25 μM) for 24 hours at 37°C. The cells were fixed in 4% (w/v) paraformaldehyde for 10 minutes, and permeabilized with 0.1% (v/v) Triton X-100 in PBS for 5 minutes. The cells were blocked for 30 minutes in PBS with 1% (w/v) BSA followed by incubation at room temperature with phalloidin-AlexaFluor-488 for 20 minutes, counterstained with DAPI for 15 minutes, and visualized under the Leica SP8 confocal laser-scanning microscope (Leica, Solms, Germany).

### Flow cytometry

Cells were incubated with mifepristone in the presence and absence of 100 ng/ml of SDF-1 for 24 hours. The cells were collected and incubated with specific antibodies against CXCR4, ICAM-1, and integrin β1, α1, α3, α5, and α6 for 30 minutes at 4°C. After washing, the cells were measured on the BD FACS Aria III system (BD Biosciences). The data were analyzed using Flow Jo sofeware (Tree Star, San Carlos, CA).

### Immunoblotting

The assay was preformed as we described previously [32]. Briefly, cells (3×10^5^ per well) were co-incubated with mifepristone in the presence and absence of 100 ng/ml of SDF-1 for 24 hours, or incubated with mifepristone alone for 24 hours followed by treatment of SDF-1 (100 ng/ml) for another 10 minutes to analyze the expression levels of Akt, ERK, p-Akt and p-ERK. After the treatments, cells were washed twice in cold PBS and lysed with RIPA buffer on ice. Lysates were normalized for protein concentration determined using the bicinchonininc acid (BCA) assay, size separated using 10% to 12% (w/v) sodium dodecyl sulfate-polyacrylamide gel electrophoresis (SDS-PAGE), electro-transferred to polyvinylidene difluoride (PVDF) membranes (Bio-Rad), blocked in 5% (w/v) skim milk in Tris-buffered saline with 0.1% (v/v) Tween-20 (TBST), and then probed using primary and HRP-conjugated secondary antibodies. Proteins were visualized using chemiluminescence by the ChemiDoc XRS System (Bio-Rad), and quantified by densitometric analysis using the Image Lab software (Bio-Rad). GAPDH was used as the internal standard for immunoblotting.

### RT-PCR

Cells (3×10^5^ per well) were co-incubated with mifepristone in the presence and absence of 100 ng/ml of SDF-1 for 24 hours. Total RNA was extracted from the cells by using TRIzol reagent (Invitrogen), and reverse transcribed with the PrimeScript^TM^ RT reagent Kit (TaKaRa, Dalian, China). The real-time experiments were conducted on the CFX96 real-time PCR system (Bio-Rad) by using the SYBR^®^ Premix Ex Taq™ (TaKaRa). The primers for MMP-9 were 5’-CTTTGACAGCGACAAGAAGTGG-3’ (forward) and 5’-GGCACTGAGGAATGATCTAAGC-3’ (reverse). The primers for MMP-2 were 5’-CAAGGACCGGTTT ATTTGGC-3’ (forward) and 5’-ATTCCCTGCGAA GAACACAGC-3’ (reverse). The primers for CXCR4 were 5’-TTCCCTTCTGGGCAGTTGAT-3’ (forward) and 5’-CCAGACGCCAACATAGACCA-3’ (reverse). The primers for GAPDH, serving as the normalization con-trols, were 5’-AGCCTCAAGATCATCAGCAATGCC-3’ (forward) and 5’-TGTGGTCATGAGTCCTTCCAC GAT-3’ (reverse). The 2^-ΔΔCt^ method was used to evaluate the relative gene expression.

### Metastatic xenograft models of human ovarian cancer

All procedures for animal experiments were carried out in accordance with the NSFC regulation, and the care and use of experimental animals was approved by the Animal Care and Use Committee of Fuzhou University to reduce the suffering and use of animals.

The *i*n vivo study was performed as we described previously [31]. Briefly, mifepristone was dissolved in normal saline supplemented with 7% (v/v) castor oil and 7% (v/v) ethanol absolute. Four-week-old female nude mice (BALB/c) were randomly assigned to four groups (*n* = 6 per group) and injected intraperitoneally with IGROV-1 cells (6×10^6^ cells / 0.2 ml / mouse). Every group of mice accepted one of the following four administrations intraperitoneally: (a) normal saline (0.2 ml) plus blank solution (0.2 ml); (b) 100 ng/ml of SDF-1 (0.2 ml) plus blank solution (0.2 ml); (c) 100 ng/ml of SDF-1 (0.2 ml) plus 5 mg/kg of mifepristone (0.2 ml); (d) 100 ng/ml of SDF-1 (0.2 ml) plus 20 mg/kg of mifepristone (0.2 ml). Each nude mouse received the pretreatment with mifepristone for three days followed by IGROV-1 inoculation, along with repeated SDF-1 and mifepristone administrations in turn every other day. Mice were sacrificed after 36 days. Volume of ascites, tumor location, and number and weight of lesions were detected to evaluate the peritoneal metastatic potential.

### Immunohistochemical analysis

The immunohistochemical assay was performed as we described previously [31]. Briefly, the tumor tissue was fixed and embedded by using formalin and paraffin wax, respectively. Tumor sections were cut at a thickness of 4 μm, deparaffinized in xylene and rehydrated in a series of ethanol solutions (100%, 95% and 75%). After antigen retrieval in citrate buffer for 10 minutes at 97°C, 3% hydrogen peroxide was used to block endogenous peroxidase activity for 10 minutes, and 10% goat serum was used to bind nonspecific antigens for 60 minutes. Subsequently, the tumor sections were incubated with the primary antibody against CXCR4 overnight at 4°C. After the detection of immuno-signals using the Vectastain ABC kit (Vector Laboratories, Burlingame, California, USA), the tumor sections were incubated with DAB, counterstained with hematoxylin, dehydrated, and then analyzed under the standard light microscopy (Zeiss).

### Statistical analysis

All statistical analyses were performed using GraphPad Prism 5. Data are represented as mean ± SD. Paired analyses were by using the Student *t* test. Multiple comparisons were by a one-way ANOVA to identify pairwise differences between more than two groups. *P* values < 0.05 were considered to indicate statistical significance.

## Abbreviations

MTT: 3-(4,5-Dimethyl-2-thiazolyl)-2,5-diphenyl-2H-tetrazolium bromide
DAPI: 4′,6-diamidino-2-phenylindole
GAPDH: glyceraldehydes-3-phosphate dehydrogenase
Akt: protein kinase B
ERK 1/2: extracellular signal-regulated kinase 1/2
MMPs: matrix metalloproteinases
COX-2: cyclooxygenase-2
VEGF: vascular endothelial growth factor
ICAM-1: intercellular adhesion molecule-1
